# Open reduction and internal fixation of quadrilateral plate fractures in the elderly: association between initial fracture pattern and outcomes

**DOI:** 10.1186/s12891-021-04002-4

**Published:** 2021-01-29

**Authors:** Haiyang Wu, Qipeng Shao, Ranran Shang, Chengjing Song, Ximing Liu, Xianhua Cai

**Affiliations:** 1 Department of Orthopaedic Surgery, General Hospital of Central Theater Command, Wuhan Clinical Medicine College of Southern Medical University, 430070 Wuhan, China; 2 Department of Orthopaedic Surgery, First Hospital of Wuhan, Hubei University of Chinese Medicine, 430022 Wuhan, China; 3 Chengjing Song Huaiyin hospital of huai an city, 223300 Huaian, China

**Keywords:** Acetabulum, Fracture fixation, Internal, Elderly, Classification

## Abstract

**Background:**

Acetabular fractures with medial displacement of the quadrilateral plate (QLP) are common in the elderly. The presence of QLP fractures greatly increase the surgical difficulty of acetabular fractures. This study aims to evaluate the clinical radiological outcomes of open reduction and internal fixation (ORIF) in QLP fractures in elderly patients and to investigate factors potentially affecting the result.

**Methods:**

We conducted a retrospective study. A series of 37 consecutive patients with acetabular fracture involving the QLP aged 60 years and older who received ORIF between January 2010 and May 2019 were included. QLP fractures were classified according to Walid’s classification system. Radiological outcomes were evaluated using Matta criteria and functional outcomes were assessed using the modified Merle d’Aubigné score. The relationships between Walid’s classification and radiological or functional outcomes were analyzed.

**Results:**

According to Walid’s classification, 18, 13, 6 were classified as QLP1, QLP2 and QLP3, respectively. The average follow-up was 35.5 ± 10.7 months. We obtained anatomic reduction in 48.6 % (18/37) of cases, imperfect reduction in 40.5 % (15/37) of cases, and poor reduction in 10.8 % (4/37) of cases. Excellent-good functional scores were found in 83.7 % (modified Merle d’Aubigné). There were no cases of screw entering the hip, pull-out and loosening or implant failure during the follow-up. Walid’s classification was positively correlated with radiological outcomes of reduction (*r* = 0.661; *P* < 0.001), and functional outcomes (*r* = 0.478; *P* = 0.003). Unsatisfactory reduction was demonstrated a correlation with the development of post-traumatic arthritis (*r* =-0.410; *P* = 0.012).

**Conclusions:**

ORIF may be suggested for quadrilateral plate fractures in the elderly. Walid’s classification system is associated with the reduction quality and functional recovery.

## Background

Acetabular fractures are relatively uncommon yet serious injuries which make up about 3–8 % of all fractures. Epidemiological investigations have shown that acetabular fractures have a bimodal distribution with respect to age that has modes at 30–40 and 70–90 years [1]. As the population ages, the incidence of osteopenic acetabular fractures resulting from low energy injuries is also increasing. Open reduction and internal fixation (ORIF) remain the preferred treatment for displaced acetabular fractures [2, 3]. However, controversies still exist regarding the optimal treatment of these fractures in older people [4, 5]. And surgical treatment represents a great challenge for orthopedic surgeons because of the decreased physiological compensatory capacity and the severe osteoporosis of the elderly [6].

Acetabular fractures in elderly patients frequently involve the quadrilateral plate (QLP), a deep and thin anatomical structure constituting the medial wall of the acetabulum. Isolated QLP fractures are rare and always associated with the anterior or posterior column fractures. Injuries resulting from the force along the femoral neck can lead to comminuted fractures of the QLP and even central dislocation of the femoral head. The QLP does not play a crucial role in the weight-bearing of the hip and is not key structure of biomechanical functionality. However, previous studies showed that a separate quadrilateral-plate component and/or central dislocation of the femoral head might adversely affect the outcomes [1, 7]. Bastian et al. [8] reported that nearly two thirds of the patients in complex fracture morphologies with medial displacement of QLP required an additional approach in addition to the modified Stoppa approach, which indicated that QLP fractures increased the complexity and difficulty of acetabular fractures surgery. The technical difficulty of ORIF of this area is predominantly due to the complicated anatomy, deep location and narrow surgical field.

This study aims to evaluate the clinical radiological outcomes of ORIF in QLP fractures in older people and to investigate factors potentially affecting the result.

## Materials and Methods

**Fig. 1:**
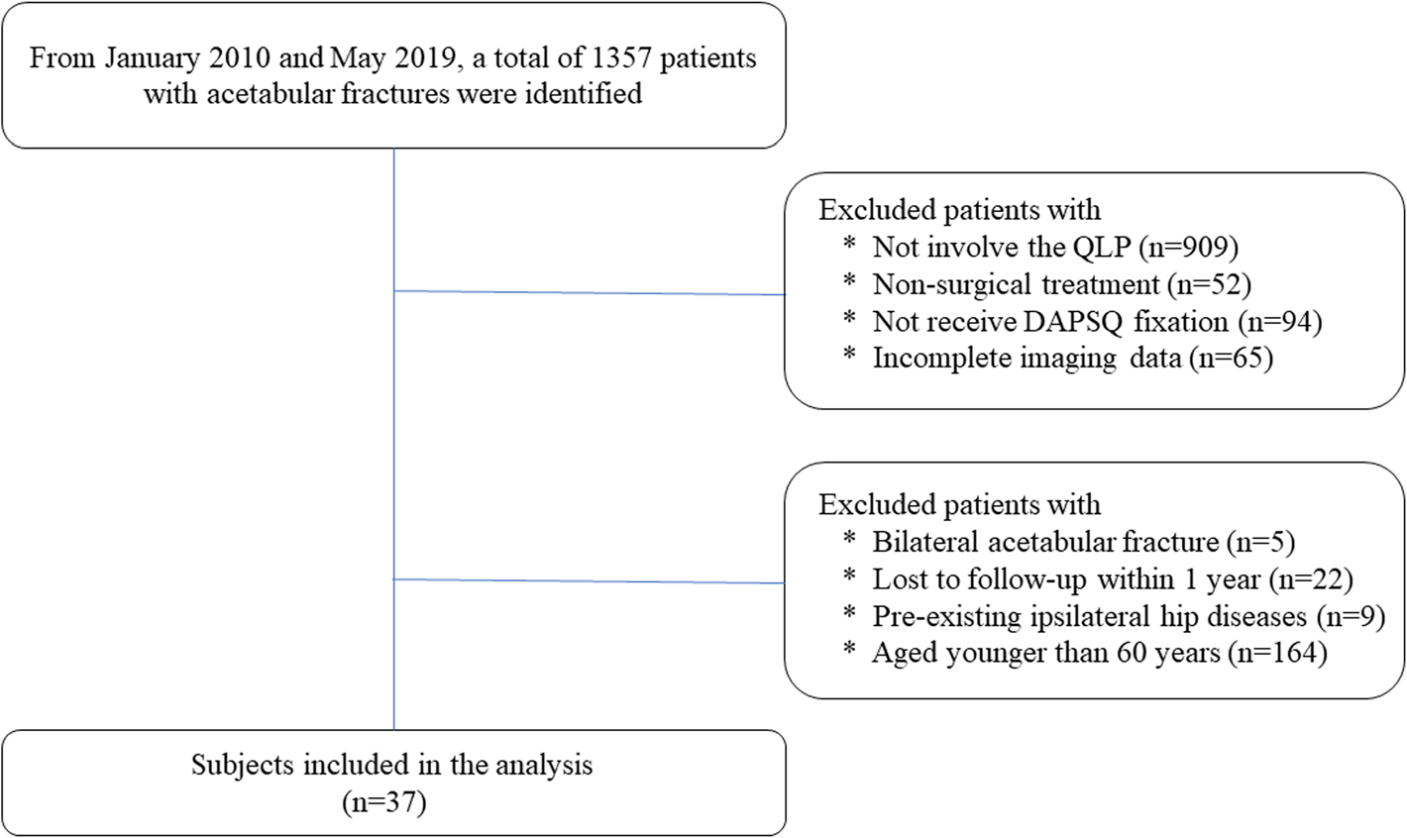
Study flow chart

Ethical approval was obtained from the ethical committee of the hospital. Between January 2010 and May 2019, patients with acetabular fractures were identified from the trauma database at our level I trauma center. Inclusion criteria consisted of all types of acetabular fractures involving the QLP, treated with Dynamic Anterior Plate-Screw system for Quadrilateral plate (DAPSQ), age older than 60 years at the time of injury, unilateral acetabular fracture, a minimum of 1-year postoperative follow up. The exclusion criteria were open or pathologic fractures, bilateral acetabular injuries, pre-existing ipsilateral hip diseases, or femoral head fracture. The flow chart of our retrospective study was illustrated in Fig. 1. A total of 37 patients with QLP fractures were included in our study eventually.

**Fig. 2:**
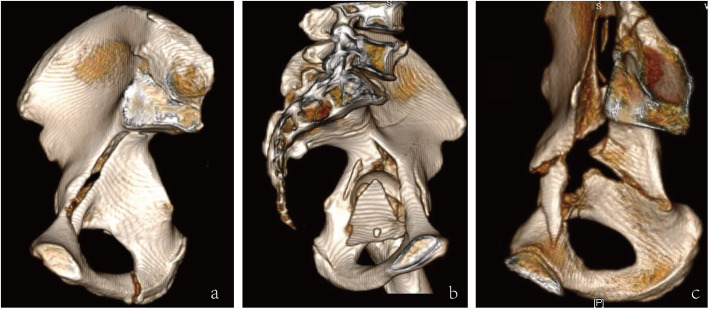
Walid’s classification of quadrilateral plate fracture. (a) QLP1, incompletely separated simple fracture; (b) QLP2, incompletely separated comminuted fracture; (c) completely separated comminuted fracture

Radiographs and medical records were collected by two investigators who were not implicated in the initial intervention. Pre-operative and post-operative radiographic analysis included Anterior-Posterior (AP) view and Judet views (iliac and obturator oblique views), along with three-dimensional (3D) CT reconstruction. Acetabular fractures were classified according to Judet and Letournel classification system [9] and QLP fractures were classified according to Walid’s classification system [10]. Walid’s classification system divides the QLP fractures into four categories as follows (Fig. 2): QLP1, incompletely separated simple fracture; QLP2, incompletely separated comminuted fracture; QLP3, completely separated comminuted fracture; QLP4, completely separated simple fracture. Pre-operative imaging evaluation should place extra attention on the “Gull sign”. When acetabular fractures involve the roof of the acetabulum, pelvic radiographs often show two typical double arc shadows on the acetabular roof, which are similar to the wings of a seagull in flight and therefore described as the “Gull sign” [11]. Medical records of all patients enrolled in this study were reviewed retrospectively, including gender, age, mechanism of injury, concomitant injuries, fracture type, and pre-existing comorbidity.

### Preoperative planning

**Fig. 3:**
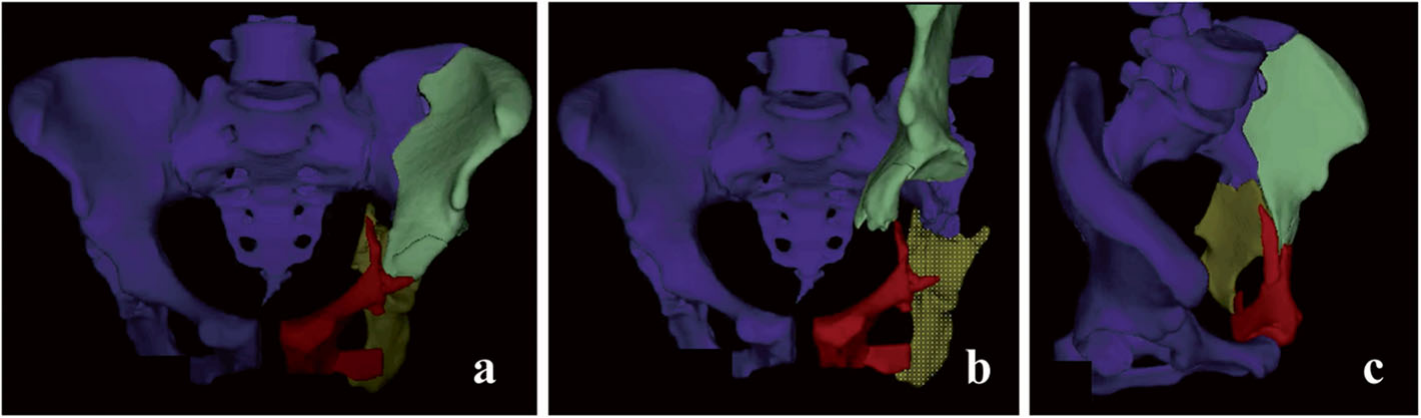
Computer-assisted preoperative planning. **a** A 3D model of acetabular fracture; **b** Select the bone fragments and perform a virtual fracture operation; **c** Fracture model after reduction

Commercially available surgical simulation software BOHOLO (Boholo Medical Technology Co., Ltd., China) was used for the pre-operative planning. All patients underwent a thin-slice pelvic CT scan (Siemens Sensation 64, Germany). The original CT images were imported as a DICOM file format into BOHOLO software. Each pelvic bones and fracture fragments were segmented virtually removing the surrounding tissue using semiautomatic thresholding tools and obtaining individual 3D digital models. All the bone fragments were considered independent and removable. And it was beneficial to accurately measure the rotation angle and displacement distance of the bone fragments, help surgeons to better understand the fracture type, as well as to simulate surgical procedures to accurately perform surgeries. Moreover, the required length of the reconstruction plate could be estimated in advance by measuring the total anatomical length of the placement trajectory of DAPSQ (Fig. 3). For comminuted and complex acetabular fractures, after obtaining informed consent from the patients, the 3D digital virtual model of the pelvis was imported into a 3D printer (Makerbot Replicator 2X, Makerbot, USA), and a rapid prototyping model of the pelvis was then printed.

### Surgical technique

All surgical procedures were performed by two experienced orthopaedic surgeons. Among them, 28 patients underwent ORIF via a classic ilioinguinal approach [12], and 9 patients was combined with the Kocher-Langenbeck (K-L) approach. The QLP fracture was reduced and temporarily fixed with various techniques, including the use of ball spike, pelvic clamp, K-wires or screws. When the “Gull sign” or the compression fracture of acetabular dome appeared on the preoperative X-ray, the compression zone could be exposed by directly prying open the fracture fragments of the QLP or indirectly fenestration and osteotomy above the acetabular dome, and restored by the implantation of autologous iliac bone or artificial bone.

**Fig. 4:**
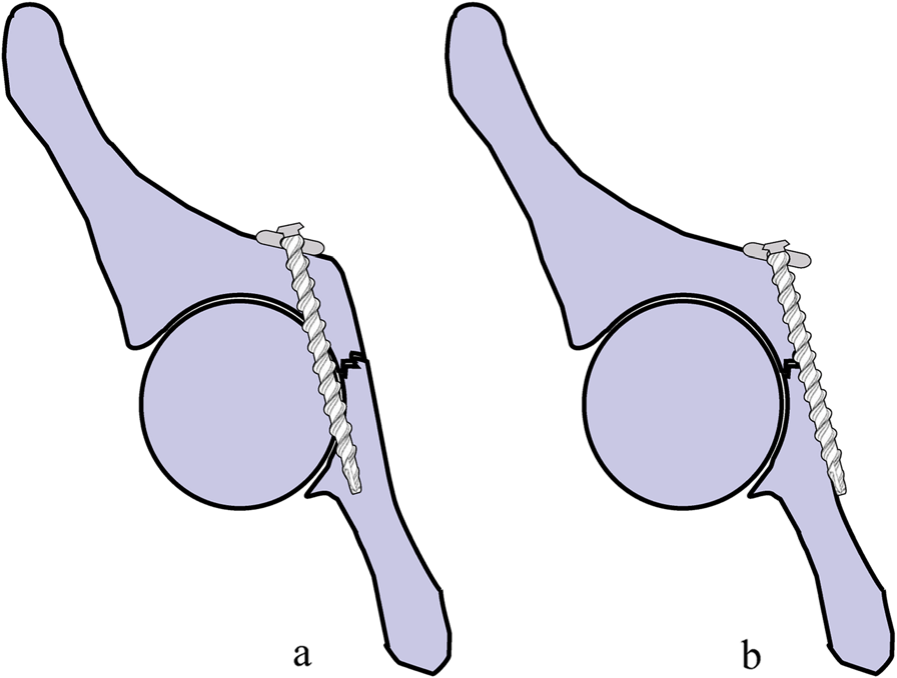
**a** Traditional fixation method: a plate placed along the medial edge of the pelvic brim with screws extending distally into the posterior column. It has a high risk of screws penetrating into the hip. **b** DAPSQ: Screws were inserted parallel to the surface of quadrilateral plate and only 1/3 to 1/2 transverse diameter of quadrilateral screws entered into the bone. This visualization method could entirely avoid the risk of hip penetration

After fracture reduction and temporary fixation, a reconstruction plate of the appropriate length was selected and shaped according to the preoperative planning and intraoperative measurements. DAPSQ plate was placed on the superior arcuate line, and the ends extended along the iliac wing and the superior pubic ramus direction, respectively. The sequence of screws placement followed certain principles described previously (Fig. 4) [13].

### Follow‐up and evaluation criteria

Radiographic and functional assessment were performed at follow-up visits through the outpatient clinic. Quality of reduction was estimated by the immediate postoperative X-ray according to the Matta radiological criteria [14], and the scores were graded as anatomic(0-1mm), imperfect (2-3mm), or poor (> 3mm) based on the millimeters of residual displacement on all views. Functional outcomes were evaluated using the modified Merle d’Aubigné score [15] at the last follow-up, and the scores were classified as excellent (18 points), good (15–17 points), fair (13 or 14 points), or poor (< 13 points). All the evaluations were performed independently by two experienced orthopedic surgeons.

### Statistical analysis

Data was collected, coded and analyzed with SPSS software (version 19.0, IBM Corp). Continuous variables were presented as the means ± standard deviations (SD). The Kolmogorov-Smirnov test (K-S) was used to test whether all continuous variables followed normal distribution. Categorical variables were presented as absolute (n) and relative frequencies (%). The Spearman rank correlation coefficient was used to measure the association between Walid’s classification and radiological or functional outcomes. The association between potential risk factors and post-traumatic arthritis was also tested. Inter-observer agreement was calculated using the kappa coefficient. *P* < 0.05 was considered statistically significant.

## Results

Patient demographics are listed in Table 1. The mean age of this group was 64.9 (SD = 3.8) years and there were 11 females (29.7 %) and 26 males. The most common fracture type was both-column fractures (40.5 %), followed by anterior column posterior hemitransverse (21.6 %). In addition, posterior wall fracture was involved in 5 cases and the rate of fractures with a Gull sign was 21.6 %.

**Table 1:** Patient demographics (*n* = 37)

Gender (n, %)	
Male	26(70.3)
Female	11(29.7)
Age,years (mean ± SD)	64.9 ± 3.8
Mechanism of injury (n, %)	
Fall from height (greater than standing)	11(29.7)
Fall (from standing height)	7(18.9)
Traffic accident	19(51.4)
Fracture side,left (n, %)	22(56.5)
Concomitant injuries (n, %)	
Head trauma	7(18.9)
Spine and sacral fracture	7(18.9)
Limb fracture	9(24.3)
Rib or clavicle fracture	6(16.2)
Dislocation of hip	13(35.1)
Others	4(10.8)
Gull sign (n, %)	8(21.6)
Pre-existing comorbidity (n, %)	
Hypertension	10(27)
Diabetes mellitus	6(16.2)
Lung disease	4(10.8)
Fracture type (*n*, %)	
Both columns	15(40.5)
Both columns + Posterior wall	3(8.1)
Anterior column	2(5.4)
Anterior column + Anterior wall	1(2.7)
ACPH	8(21.6)
T type	3(8.1)
Transverse and posterior wall	2(5.4)
Transverse	3(8.1)

*ACPH* anterior column and posterior hemitransverse

Treatment was standardized with a mean interval from injury to surgery of 10.5 days with a range of 6–17 days. In regard to surgical approach, 28 (75.7 %) patients were treated with single ilioinguinal approach, and 9 (24.3 %) patients (5 cases of posterior wall fracture, 3 cases of both-column fracture and 1 case of T type fracture) were combined with a K-L approach. Surgical time averaged 256.9 minutes (SD = 58.9). Intraoperative blood loss averaged 1029.7 mL (SD = 442.8). Eventually 86 % of the cases received a blood transfusion with an average of 662.2 mL (SD = 486.7). The mean duration of hospital stay was 26.1 days (SD = 6.4). No intra-operative complications were observed. Surgery details of all included patients were listed in Table 2.

**Table 2:** Surgery details, followed-up time and complications (*n* = 37)

Time to surgery, days (mean ± SD)	10.5 ± 3.1
Surgical approach (*n*, %)	
Ilioinguinal	28(75.7)
Ilioinguinal + Kocher-Langenbeck	9(24.3)
Surgical time, min (mean ± SD)	256.9 ± 58.9
Blood loss, mL (mean ± SD)	1029.7 ± 442.8
Blood transfusion, mL (mean ± SD)	662.2 ± 486.7
Hospital stay time, days (mean ± SD)	26.1 ± 6.4
Followed-up time, years (mean ± SD)	35.5 ± 10.7
Complications (*n*, %)	
Deep venous thrombosis	1(2.7)
Lateral femoral cutaneous nerve injury	3(8.1)
Superficial wound infection	1(2.7)
Posttraumatic arthritis	7(18.9)
THA	2(5.4)

*THA* total hip arthroplasty

Kappa analysis showed a high consistency between the two senior surgeons for the subjective score with Kappa coefficient of 0.87. According to the definition of the quality of the reduction, 18 patients (48.6 %) showed anatomical reduction, 15 (40.5 %) had an imperfect reduction, and 4 (10.8 %) had a poor reduction.

**Fig. 5:**
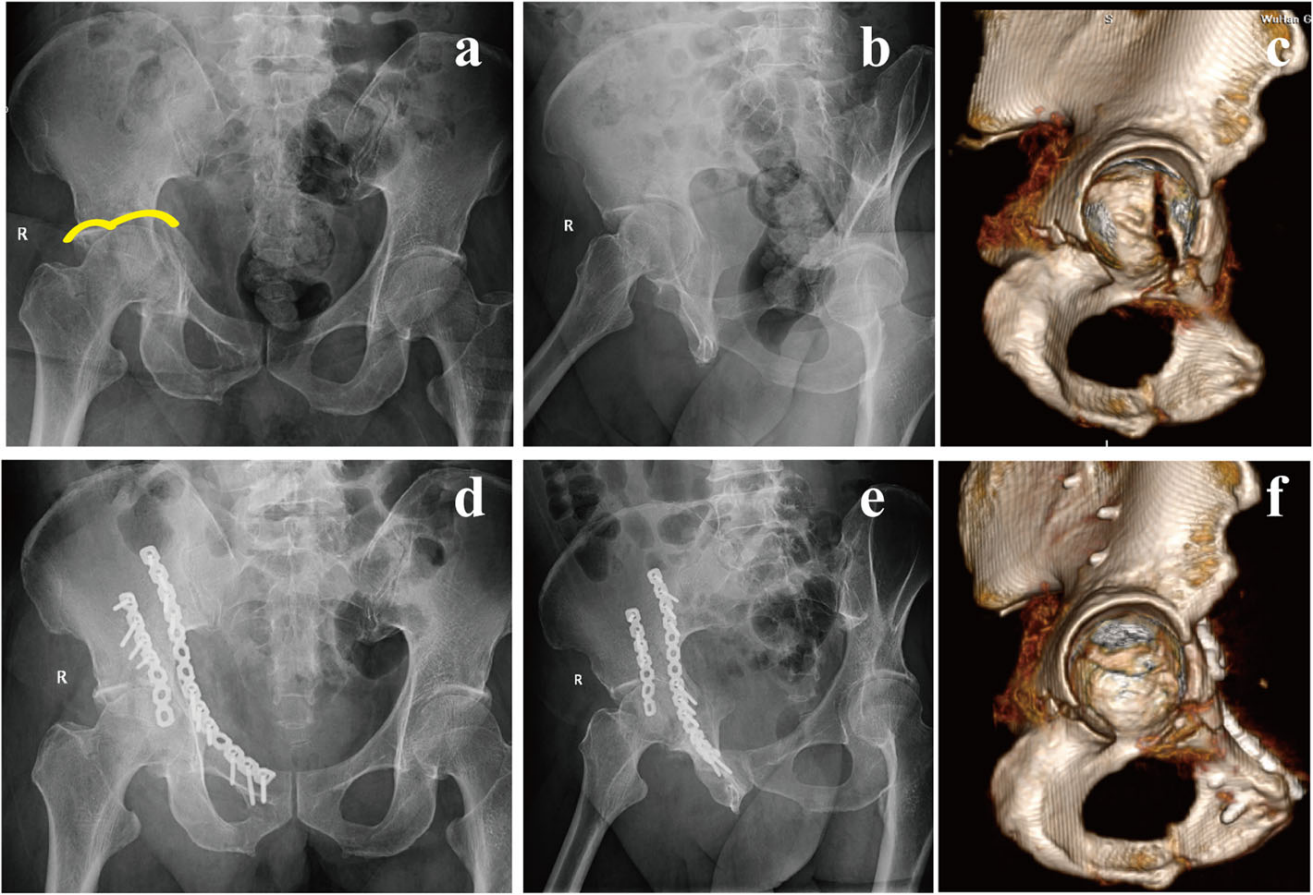
A 61-year-old man presented with acetabular fracture of the right acetabulum. Preoperative AP view (**a**) showed a typical feature of “Gull sign”. Judet view (**b**) and 3D CT reconstruction (**c**) showed that the quadrilateral plate was separated from the anterior column and partially attached to the posterior column, which was classified as QLP1. ORIF was performed via a single ilioinguinal approach, and the compression fracture of acetabular dome was managed with artificial bone grafting. Post-operative X-rays (**d, e**) and 3D view (**f**) showing an anatomical reduction

Patients were followed-up for more than 12 months with a mean follow-up period of 35.5 months (SD = 10.7). There were no cases of screw pull-out, screw loosening or implant failure during the follow-up. At the last follow-up, the mean modified Merle d’Aubigne score was 16 (range 10–18), categorized as excellent in 16 cases (43.2 %), good in 15 cases (40.5 %), fair in 4 cases (10.8 %), and poor in 2 cases (5.4 %). Postoperative complications were presented in Table 2. And one typical case was shown in Fig. 5.

According to Walid’s classification, 18 patients were classified as QLP1, 13 patients were classified as QLP2 and 6 patients were classified as QLP3. Walid’s classification was strongly positively correlated with radiological outcomes of reduction (*r* = 0.661; *P* < 0.001), and moderately positively correlated with functional outcomes (*r* = 0.478; *P* = 0.003) (Table 3).

**Table 3:** The relationships between Walid’s classification and radiological or functional outcomes

Walid’s classification	Radiological outcomes			Functional outcomes			
	Anatomical	Imperfect	Poor	Excellent	Good	Fair	Poor
QLP1(18)	15	3	0	12	5	0	1
QLP2(13)	2	9	2	3	8	1	1
QLP3(6)	1	3	2	1	2	3	0
Total (37)	18(48.6)	15(40.5)	4(10.8)	16(43.2)	15(40.5)	4(10.8)	2(5.4)

*QLP* quadrilateral plate

In addition, the quality of reduction was strongly positively correlated with the functional outcomes (*r* = 0.701; *P* < 0.001). Unsatisfactory reduction was also demonstrated a correlation with the development of post-traumatic arthritis (*r* =-0.410; *P* = 0.012). The associations between Gull sign or Walid’s classification and post-traumatic arthritis revealed no statistically significant differences (*P* = 0.644 and *P* = 0.133, respectively). The relationships between Letournel classification and radiological or functional outcomes were also analyzed. The results showed that no significant correlation existed between both columns, ACPHT or involved posterior wall and radiological or functional outcomes (*P* all > 0.05) (Table 4).

**Table 4:** The relationships between Letournel classification and radiological or functional outcomes

Letournel classification	Radiological outcomes		Functional outcomes	
	*r*	*P*	*r*	*P*
Both columns	0.237	0.159	0.318	0.055
ACPHT	0.116	0.496	0.290	0.082
Involved posterior wall	0.229	0.172	0.177	0.296

*ACPH* anterior column and posterior hemitransverse

## Discussion

The management of acetabular fractures in elderly is controversial. Both conservative and surgical treatment have been reported to be risky and potentially unsuccessful [4, 5]. The current, commonly used surgical options for the elderly with acetabular fractures include minimally invasive percutaneous internal fixation [16], ORIF [17], or hip replacement [18]. Anatomic reduction and stable fixation are primary goals of ORIF. The attention of many orthopaedic surgeons today is not only aimed at technological innovations from fixation devices and surgical approach but also at improving the effectiveness of those already used in the clinics. In most cases, as shown in Fig. 4, placing a reconstruction plate along the pelvic brim of the anterior column with screws extending distally into the posterior column can provide adequate stability when used in non-osteoporotic bone, but at the risk of screws penetrating into the hip.

DAPSQ was designed based on the traditional anterior reconstruction plate [13, 19]. Several biomechanical experiments have confirmed its reasonable mechanical stability [20, 21]. We consider that this technique is more suitable for acetabular fractures involving the anterior column, including both-column fractures and transverse fractures mainly with anterior column displacement, certain types of T-shaped and ACPHT fractures [19]. Although acetabular fractures involving the anterior column are the most common fracture types in the elderly, DAPSQ is not suitable for all types. For delayed acetabular fractures, acetabular posterior wall fractures, and acetabular fractures mainly involving posterior column, fixation of the posterior column with reconstruction or locking plates through a posterior approach is often needed.

The results of the present study show that 75.7 % of patients were treated through a single ilioinguinal approach, and an additional Kocher-Langenbeck approach was required in 9 patients. Reduction after operation was considered anatomical in 48.6 % of the patients, and 83.7 % of them had excellent or good functional outcomes. Peter et al. [3] reported 84.6 % excellent and good functional outcome with the L-shaped buttress plate to treat QLP fractures in 13 elderly patients, which was similar to results seen in our study. Another study by Laflamme et al. [22]. included 21 patients with osteopenic QLP fractures. They found that 52.4 % (11/21) of patients obtained anatomic reduction and 92.9 % with excellent and good functional outcomes by using an infra-pectineal buttress plate. These results differ from our data and may be attributed to several factors such as different age range (55–82 years) and choice of surgical approach (modified Stoppa).

Recently, Herman et al. [23] proposed a new classification scheme for acetabular fractures, identifying three different types based on the displacement vector and the fractured anatomic structures. According to this classification, the medial displacement of the QLP was regarded as a hallmark of the superomedial displacement vector. Walid et al. [10] further divided the QLP fractures into four categories. As far as we know, this is the first study to explore the relationship between Walid’s classification and radiological or functional outcomes.

The results demonstrated that Walid’s classification was strongly and positively correlated with radiological outcomes of reduction and moderately and positively correlated with functional outcomes. Walid’s type QLP1 fracture was associated with better radiological and functional outcomes, while type QLP3 fracture was associated with poorer outcomes. The findings suggest that Walid’s classification may reflect the severity of acetabular fracture to some extent and mainly relate to the degree of comminuted fracture of the QLP. Thus, Walid’s classification may guide the selection of an appropriate surgical approach and fixation strategy. The relationships between Letournel classification and radiological or functional outcomes were also analyzed. The results showed that no significant correlation existed between both columns, ACPHT or involved posterior wall and radiological or functional outcomes. This result may reveal that preoperative assessment of the fracture pattern according to the Walid classification is better than the classic Letournel classification in predicting outcomes, and possibly modifying the surgical management. However, it is worth noting that there are still some limitations to consider when interpreting these findings. For example, Walid’s type QLP4 fracture seems to be a theoretical possibility, and has not yet been reported [10].

Previous studies also have shown that “Gull sign” in preoperative X-ray may be an important sign of poor functional outcomes in the elderly with acetabular fractures [24]. Unlike their study, our study did not find a correlation between “Gull sign” and post-traumatic arthritis. In accordance to our view, Carroll et al. [25] and Zhuang et al. [11] also considered that “Gull sign” had no correlation with secondary THA. Even if the “Gull sign” appeared in preoperative X-ray of the elderly patients, satisfactory outcomes can be obtained by sufficient reduction and bone grafting of the compressed dome.

This study also introduced virtual technology into the preoperative planning of acetabular fractures in the elderly. This technique can help surgeons to understand the fracture pattern, rotation angle and displacement distance of fragments, and simulate the reduction process of fracture on the computer before operation. And design the optimal reduction sequence and further provide a reference for the selection and measurement of the plate. Many scholars have advocated the application of virtual and 3D printing technology in the treatment of pelvic and acetabular fracture [26, 27]. However, it should be noted that the basis of computer assisted surgery is the 3D model of acetabular fracture, either through virtual technology or 1:1 printed 3D fracture model. However, the technology is limited to the bone and does not include soft tissue or ligaments, thus being still quite different from the real setting.

Our study has some limitations due to its retrospective design and limited number of patients. Another restriction is the absence of a comparative group. Further randomized controlled trials and larger numbers of participants will be necessary to confirm these findings. In addition, although all surgical procedures in this study were performed by two surgeons, the technique was well-standardized to eliminate the effects of different surgeons on outcome variables.

## Conclusions

ORIF is still the preferred treatment for displaced acetabular fractures in the elderly. DAPSQ is an optional and effective technique for the treatment of acetabular fracture involving the QLP and has the advantages of avoiding screws penetrating into the hip. Besides, Walid’s classification system is associated with the reduction quality and functional recovery. QLP fractures classified as QLP3 may need a more precise and appropriate operative strategy to improve the success of surgery.

## Data Availability

The datasets generated and/or analyzed during the current study are available from the corresponding author by reasonable request.

## Abbreviations

DAPSQ: Dynamic Anterior Plate-Screw System for Quadrilateral plate
QLP: Quadrilateral plate
AP: Anterior-Posterior
CT: Computed Tomography
3D: Three-Dimensional
ACPH: Anterior Column Posterior Hemitransverse
THA: Total Hip Arthroplasty
